# The Use of Whey in Infant Feeding

**Published:** 1909-06-26

**Authors:** 


### Diseases of Children.

### THE USE OF WHEY IN INFANT FEEDING.

The value of whey in the dietary of delicate infants is so great that it is worth while to take the trouble to understand its composition and various methods of preparation. Strictly it is the fluid which is squeezed out of the curd produced by the coagulation of milk by rennet—that is, it is milk deprived of casein and fat, for the fat is entangled in the meshes of the curd and removed with it. Frequently it is enriched by breaking up the curd with a fork and forcing it through fine muslin. Such whey contains a considerable amount of fat and casein, the latter finely broken up and not reuniting into tough coherent curds in the infant's stomach. The estimations of the percentage of albumin in whey and milk vary considerably, as is seen in the following table:—

Percentage of Albumin in Whey.

| | | | |
|---|---|---|---|
| Lehmann ... | ... 0.30 | Hammarsten ... | 0.86 |
| Raudnitz ... | ... 0.30 | Smetham... | ... 0.88 |
| Hoppe-Seyler ... | 0.30 to 0.50 | White and Ladd | 0.90 to 1.02 |
| König ... | ... 0.53 to 0.86 | Veith... ... | ... 0.92 |
| Van Slyke... | ... 0.66 | U. S. Depart. of Agriculture ... | 0.93 |
| Gorup-Besanez... | 0.75 | Fleischmann ... | 1.00 |
| Wynter Blyth ... | 0.77 | | |

Cautley obtained the high average of 1.41 per cent., and casein 2.61 per cent. in milk. The variations in the results do not enable us to fix on an absolute figure as representing the invariable percentage, but it is fairly safe to assume that the fluid contains about 0.85 per cent. of albumin. In addition it contains the sugar and salts in the same proportions practically as in the original milk.

In curdling milk by rennet it is important to remember that, if the whey is to be used as a diluent of ordinary milk, the rennet ferment must be destroyed by heat. Otherwise on adding it the resulting mixture curdles. Similarly, if cream is added to whey, more or less curdling is produced, varying in degree according to the richness of the cream in fat and deficiency in casein. Thus, little curdling occurs if a centrifugal cream containing 40-50 per cent. fat is added, not sufficient to be of any importance, but it may be somewhat troublesome if gravity cream is used. To destroy the ferment the whey must be heated to 150° F. Higher temperatures are likely to coagulate the albumin and perhaps make it less digestible.

Whey can be prepared from fresh or from skimmed milk, the former by preference. Prime Bristol rennet, essence of rennet, liquid rennet, Benger's or Lazenby's rennet, or junket tablets are added to the milk, which is then warmed to about 100° F. for half an hour. It is allowed to stand until curdled and the whey has separated out as the curd contracts, forming a somewhat turbid fluid which can be strained off. Liquid preparations of rennet vary much in strength, and the glycerine with which they are made may not be pure. Beauchamp Hall has advised the use of a powdered form of rennet, purified and standardised, and made into tablets. Each tablet contains enough rennet to curdle half a pint of milk—sod. bicarb. gr. 1½, calcium phosphate gr. ½, and lactose gr. 5. The ferment acts better in the presence of the calcium salt, and the bicarbonate neutralises lactic acid, if present. Ten ounces of milk should yield seven and a half of whey.

A simple method of preparing milk so that it contains only half the normal percentage of casein, the other constituents remaining about the same, is to stand the milk in a cool place for three hours, separate the milk by skimming, divide the milk into two halves and curdle one by rennet. Strain off the whey and heat it to 150° F. Then mix the cream, whey, and the other half of the milk. As a rule it is simpler to add cream only to the whey, and then, according to the needs of the child and its digestive capacity, add milk in gradually increasing quantities. The addition of sugar is necessary in all cases. *Tartrated whey*, prepared after a method devised by Still and Myers, is a cheaper food than that made by rennet. Eight grains of tartaric acid, dissolved in half a drachm of water, are added to half a pint of milk which has been heated until it just begins to bubble. The mixture is kept simmering for five minutes, at the end of which time it is curdled and can be strained through butter muslin. According to Still, the whey is faintly acid to litmus, and contains protein 0.58 per cent., fat 1.2 per cent. The proportion of protein seems rather low. The addition of acetic acid causes no further coagulation, and this whey can be added to milk without causing curdling. *White wine whey* or *sherry whey* is made by the action of the acetic and tartaric acids present in the wine. Myers and Still have pointed out that it is better to use a cheap cooking sherry in preference to a more expensive drinking sherry. The cheaper wine contains less alcohol and more acid, so less is required for curdling the milk, and the resulting whey is weaker in alcoholic strength; an important feature if it is to be used as an article of diet for some time. Bring ten ounces of milk to a boil, add two and a half ounces of the cooking sherry, and again heat it until the mixture boils up. Then stand it for three minutes and strain through butter muslin. According to Still, the alcoholic strength is such that one ounce of the whey is equivalent to 25 minims of brandy. It must be regarded as an emergency food. Whey can also be prepared by boiling a pint of milk with two teaspoonfuls of lemon juice.

It must not be supposed that whey is a perfect food, even if cream and sugar are added. Undoubtedly some infants make very rapid progress on the mixture for a time, but it has seemed to the writer that its deficiency in casein is a distinct drawback. There is a certain amount of evidence that albumin cannot replace casein in infant feeding. Possibly the albumin is required for nutrition, and casein for growth. In delicate babies, or those with gastro-intestinal disorders, whey, even if diluted with equal parts of water, may not be digested.

